# Adipocytokine Profile, Cytokine Levels and Foxp3 Expression in Multiple Sclerosis: a Possible Link to Susceptibility and Clinical Course of Disease

**DOI:** 10.1371/journal.pone.0076555

**Published:** 2013-10-03

**Authors:** Solaleh Emamgholipour, Seyede Mahdieh Eshaghi, Arash Hossein-nezhad, Khadijeh Mirzaei, Zhila Maghbooli, Mohammad Ali Sahraian

**Affiliations:** 1 Department of Clinical Biochemistry, School of Medicine, Tehran University of Medical Sciences, Tehran, Iran; 2 Department of Medicine, Section of Endocrinology, Nutrition, and Diabetes, Vitamin D, Skin and Bone Research Laboratory, Boston University Medical Center, Boston, Massachusetts, United States of America; 3 Tehran University of Medical Sciences, Tehran, Iran; 4 School of Public Health and Institute of Public Health Research; 5 Department of Neurology, Sina MS Research Center, Sina Hospital, Tehran University of Medical Sciences, Tehran, Iran; University of Lyon, France

## Abstract

**Background:**

Adipocytokines may be involved in multiple sclerosis (MS) as well as other autoimmune and inflammatory-related diseases. This study aims to compare levels of resistin, visfatin and leptin in three subgroups of MS patients with healthy subjects and also to study their relationship with Foxp3 expression and levels of several pro-inflammatory mediators such as interleukine-1 β(IL-1 β),tumor necrosis factor-α (TNF-α) and human sensitive C-reactive protein (hs-CRP).

**Methods:**

A total of 391 subjects including 200 healthy controls and 191 MS patients were recruited for this case-control study. Circulating adipocytokines and inflammatory mediators were measured using immunoassay methods. Foxp3 gene expression in peripheral blood mononuclear cells (PBMC) was determined by quantitative real-time PCR. Fat tissue mass was evaluated by using dual energy X-ray absorptiometery (DEXA).

**Results:**

A significant difference was observed in levels of inflammatory mediators, adipocytokines, Foxp3 gene expression and adipose tissue mass between MS patients and healthy controls. All adipocytokines were positively correlated with levels of inflammatory mediators and negatively correlated with Foxp3 expression in MS patients. In controls, there were positive correlations between circulating leptin and resistin with TNF-α and IL-1β in subgroup analysis, the highest levels of TNF-α, IL-1β, hs-CRP, resistin and leptin were observed in primary progressive-MS (PP-MS) patients. Also, expression of Foxp3 and levels of visfatin in relapsing remitting-MS(RR-MS) patients were higher compared with the other subgroups.

**Conclusions:**

Our findings suggest the potential role of adipocytokines in pathogenesis and severity of MS. Notably, the relationship of adipocytokines levels with inflammatory cytokines as well as clinical features of MS could be considerable in translational medicine and biomarker studies.

## Introduction

Multiple sclerosis (MS), the most common cause of neurological disability in young people and middle-aged adults, affects up to 2 million people worldwide[1].

Importantly, it represents a considerable heterogeneity regarding clinical course, immunological features, pathogenesis and also imaging appearances[2].

Although, the etiology of MS is not fully understood, available evidence indicates that immune system plays an important role in pathogenesis of MS [3-5].

Increased production of Th1 cell-derived cytokine (IFN-γ, IL-12 and TNF-α) and Th17 cell-derived ones (IL-17 and IL-6), and reduced number and activity of regulatory T (Treg) cells appears to play an important role in this regard [4,6-8].

Notably, Treg cells by expression of the forkhead transcription factor (Foxp3) have a vital role in promoting and maintaining of self-tolerance, thereby preventing chronic inflammatory diseases and autoimmune diseases [9-11].

Several studies have demonstrated that alternation in Treg cell function and amount of pro inflammatory cytokines, determines disease susceptibility and/or affects relapse and remission phase in MS[12-15].

Recent limited studies demonstrated that adipocytokines including resistin, leptin and visfatin may be important in MS pathogenesis [16-18].

 Leptin, an adipocyte-derived cytokine, belongs to the type I cytokine superfamily with structural similarity to IL-6, IL-12, IL-15 and granulocyte colony-stimulating factor [19].

Leptin-deficient (ob/ob) mice showed resistance to the autoimmune disorders including Ag-induced arthritis [20], experimental colitis [21], type 1 diabetes [22], and experimental autoimmune encephalomyelitis (EAE) [23]. 

Analysis of transcriptional profiling in MS lesions revealed that leptin expression is increased at the site of inflammation in brain[24]. 

Resistin, known as adipocyte-secreted factor (ADSF) or found in inflammatory zone 3 (FIZZ3), is secreted from immunocompetent cells, including macrophages and mononuclear cells in humans. Several lines of evidences support its engagement in inflammatory conditions *in vitro* as well as *in vivo* (reviewed in 25).

Visfatin, an adipokine secreted from visceral fat, originally called pre-B cell colony-enhancing factor (PBEF) by virtue of its function as a putative cytokine in B cell development. This protein was also considered as the rate-limiting step in a salvage pathway of nicotinamide adenine dinucleotide (NAD) biosynthesis and thus named nicotinamide phosphoribosyltransferase (NAMPT)[26].

The importance of NAD metabolism in the development of both T and B lymphocytes, the enhancement of NAMPT upon T cell activation and also decrease in the clinical manifestations of EAE using pharmacological inhibitor of NAMPT, provided evidence for the significance of visfatin/NAMPT in MS pathogenesis [27,28].

In addition, *in vivo* and *in vitro* studies demonstrated that expression of leptin, resistin and visfatin is up-regulated during activation of immune cells such as monocytes, macrophages, dendritic cells, B and T lymphocytes and also these proteins beyond their role in metabolism can influence diverse aspects of inflammatory and immune pathways, including regulation of cytokine secretion, macrophage and neutrophil activation [29-32].

Although, vigorous efforts have been made to delineate the role of adipocytokines in metabolic syndrome, diabetes, obesity and cardiovascular diseases , there is still a great deal of uncertainty about their roles in autoimmune diseases such as MS. Thus far, no study evaluating the association of circulating adipocytokines levels with various course of MS, has been undertaken. In addition, many of the molecules currently in focus as therapeutic targets for the treatment of MS are still associated with regulation of immune system function, hence, more detailed understanding of the immune and inflammatory pathways regulated by adipocytokines and apparently complex cross-talk between them will open up new prospects for therapeutic approaches in MS.Therefore, the current study was designed to simultaneously measure the concentrations of serum resistin, visfatin and leptin in three subgroups of MS patients compared with healthy subjects and also to study their relationship with FoxP3 expression as well as levels of several pro-inflammatory cytokines such as IL-1 β, TNF-α and hs-CRP.

## Materials and Methods

### Subjects

Totally, 391 subjects, including 191 MS patients and 200 age- and gender-matched healthy individuals were recruited for this case - control study.

The MS patients were recruited from the Iranian Multiple Sclerosis Society and the diagnosis was confirmed by a neurologist based on the McDonald’s criteria. Patients were divided into 3 subgroups according to clinical patterns at onset, namely: relapsing remitting-MS (RR-MS; n=118, 62%), secondary progressive-MS (SP-MS; n=42, 22%) and primary progressive - MS (PP-MS; n=31, 16%). The inclusion criteria were defined as: clinically definite MS with a disease duration of 1-10 years and an Expanded Disability Status Scale (EDSS) score ≤ 5.5, age between 20-45 years. Participants with a history of cardiovascular disease, hypertension, diabetes mellitus (type I and II), thyroid disease, current smoking, hepatic or renal disease, malignancy and acute or chronic infection (according to the patient’s data base) were excluded from the study. The control group was selected among the general population in the same geographical areas as patients. It is worth bearing in mind that the differences in age and gender were not significant between the patients and controls. The study was approved by the local ethics committee of Endocrinology and Metabolism Research Institute of Tehran University of Medical Sciences. Informed written consent was signed by all subjects before their participation in the study. It should be noted that none of the patients had received immunosuppressive or immunomodulatory therapy for at least three months prior to sample collection.

### Laboratory measurements

Venous blood was collected following an overnight fasting in coagulant containing vacutainers.

Then, samples were centrifuged and serum separated and stored at -80 °C until assayed.

Levels of IL-1β, TNF-α and hs-CRP in serum samples were determined as previously described [33]. Briefly, Levels of IL-1β and TNF-α in serum samples were determined by immunoassay (ELISA) using R&D system kit (R&D system, USA) and the serum hs-CRP concentration was measured by imonoturbidimetric assay (High sensitivity assay, by Hitachi 902). The intra and inter-assay coefficients of variation (CVs) for IL-1β and TNF-α were 4.8%, 4.1%, 4.8% and 6.1%, respectively.

The measurement of serum concentration of visfatin was performed as previously described method [17] using Human visfatin ELISA kit (AdipoGen Pharmaceuticals, Belmont, Seoul, Korea). The interassay variability and the intra-assay variability was 5.5% and 4.3% respectively.

The serum resistin concentration was measured by ELISA method (LINCO Research Inc, St Charles, MO, USA). The sensitivity of the assay was 0.095 ng/ml and the inter- and intra-assay coefficients of variation were 5.9% and 5.8%, respectively.

The serum concentration of leptin was determined by immunoassay (ELISA) using a R&D system kit (R&D system, USA). The Minimum detectable concentration was 0.0078 ng/ml, intra- and inter-assay coefficients of variation (CV) were 3.1% and 4.4 %, respectively. 

### Fat mass and Body Mass Index (BMI) measurements

The weight (to the nearest 0.1 kg) and height (to the nearest 0.1 cm) were measured following an overnight fasting, in light clothing and barefoot. BMI was calculated as body weight (kg)/height^2^ (m^2^). For all participants, fat tissue mass was measured using dual energy X-ray absorptiometry (DEXA) (Lunar-DPX,USA) at lumbar spine (in the region of vertebrae L2-L4) and hip regions as described. The coefficient of variation for longitudinal fat mass measurements in the DEXA machine averaged at 1.04%. 

### Foxp3 gene expression

Foxp3 gene expression was evaluated by quantitative real-time PCR (RT-PCR) in a Step-One-Plus TM real-time (ABI Applied Biosystems) as previously described[12]. Briefly, total RNA was extracted from PBMC using TRIzol reagent (Invitrogen) and 1 μg of total RNA was reverse transcribed using Revert Aid First Strand cDNA Synthesis Kit (Fermentas Life Science, Vilnius, Lithuania), according to the manufacturer's instructions. 

Approximately 50 ng of the resulting cDNA was amplified in a total volume 20 μL using 2X SYBR Premix Ex Taq™ Master Mix (Takara, Japan), and 10 μM of each primer.

All assay efficiencies were monitored using a standard curve. The housekeeping gene β-actin served as an internal control. 

### Statistical analysis

Numerical variables were reported as the mean ± Standard deviation and categorical variables were presented as percentage. All of the statistical analyses were performed using the Statistical Package for the Social Sciences (SPSS, version 15, Chicago, IL, USA). Student *t*-test and analysis of variance (ANOVA) were used to compare the means and Chi-square test was used to compare the qualitative variables. Pearson correlation was used to investigate the correlation between the quantitative variables. Differences were considered statistically significant at level of P≤0.05.

## Results

Anthropometric and clinical characteristics of MS patients and control subjects are presented in table 1.

**Table 1:** Demographic and characteristics of study population.

Characteristics	participants		
	MS (n=191)	Controls(n=200)	P value
Age(years)	35.16±8.64	36.11±8.43	0.3
Gender (female)	129(67.5%)	134(67%)	0.7
Height(cm)	163.97±8.32	163.87±8.56	0.7
Weight(kg)	63.8±12.81	64.3±12.71	0.6
BMI(kg/m^2^)	24.41±4.35	24.68±4.75	0.6
Fat mass L2-L4 (%)	18.26±8.78	22.57±9.26	0.01

BMI: body mass index.

Values are expressed as mean ± SD. Comparison of variables means in two groups performed by Student’s t test.

There was no significant difference between the two study groups regarding age, sex, height, weight and body mass index, however, adipose tissue mass percentage significantly decreased in patient group as compared with control subjects (18.26±8.78 vs. 22.57±9.26, p<0.05).

Serum levels of TNF-α, IL-1β and hs-CRP as inflammatory mediators in MS patients were significantly higher in comparison with control subjects (Table 2).

**Table 2:** Serum levels of inflammatory mediators and relative expression of foxp3 in peripheral blood mononuclear cells of study population.

Characteristics	participants		
	MS (n=191)	Controls(n=200)	P value
TNF-α (ng/ml)	1.52±0.85	0.76±0.55	0.01
IL-1β (ng/ml)	0.93±0.64	0.53±0.45	0.01
hs-CRP (mg/l)	1.72±1.13	0.73±0.47	0.02
Foxp3 relative expression	0.21±0.1	0.31±0.08	0.001
Visfatin(ng/ml)	10.55±4.32	5.34±2.27	0.01
Leptin (ng/ml)	13.81±10.22	8.21±5.63	0.01
Resistin(ng/ml)	14.65±5.23	9.44±4.12	0.01

TNF-α: tumor necrosis factor-alpha; IL-1β: interleukin-1 beta; hs-CRP: high sensitivity C-reactive protein; Foxp3: forkhead box P3. Values are expressed as mean ± SD. Comparison of variables means in two groups performed by Student’s t test.

Also, serum levels of resistin, visfatin/Nampt and leptin were significantly higher in MS patients compared with healthy subjects. 

As shown in table 2, Foxp3 mRNA expression in MS patients is significantly decreased in patients with MS compared to healthy controls 

In subgroup analysis (table 3), there was no significant difference between RR-MS, SP-MS and PP-MS, regarding age, gender, height, weight, BMI and adipose tissue mass. The serum levels of IL-1β, and hs-CRP were significantly elevated in PP-MS patients compared to those of RR-MS. In addition, PP-MS patients showed higher serum levels of TNF-α, compared with two other subtypes, although this difference was not statistically significant.

**Table 3:** Comparison of demographic characteristics, FoxP3 expression and circulating inflammatory mediators in MS patients respect to clinical subtypes.

	PPMS(n=31)	SPMS(n=42)	RRMS(n=118)
Age(years)	36.73±9.05	35.65±8.87	34.57±8.31
gender(female)	24(77.4%)	29(69%)	76(64.4%)
Height(cm)	164.42±8.65	164.89±8.94	163.55±8.58
Weight(kg)	63.85±13.11	62.86±13.32	64.12±12.71
BMI(kg/m^2^)	23.73±4.35	23.08±5.13	24.13±4.32
Fat mass L2-L4 (%)	18.04±9.06	17.67±9.11	18.54±8.97
TNF-α (ng/ml)	1.67±1.03	1.44±0.96	1.5±0.55
IL-1β (ng/ml)	1.36±0.92	0.98±0.83	0.79±0.56^a^
hs-CRP (mg/l)	2.21±1.44	1.74±0.97	1.58±0.88^a^
Foxp3 relative expression	0.18±0.09	0.19±0.09	0.22±0.11
Visfatin(ng/ml)	9.11±4.61	8.04±4.12	11.83±5.14^a,b^
Leptin (ng/ml)	15.23±11.41	14.71±10.32	10.25±9.46 ^a^,^b^
Resistin(ng/ml)	15.18±5.12	14.19±5.16	14.58±4.81

RRMS: relapsing remitting multiple sclerosis; SPMS: secondary progressive multiple sclerosis; PPMS: primary progressive multiple sclerosis ; BMI: body mass index; TNF-α: tumor necrosis factor-alpha; IL-1β: interleukin-1 beta; hs-CRP: high sensitivity C-reactive protein; Foxp3: forkhead box P3.

Values are expressed as mean ± SD. Comparison of variables means in two groups performed by Student’s t test.

a Significant difference between RRMS vs PPMS (p<0.01)

b Significant difference between RRMS vs SPMS(p<0.010)

Circulating levels of visfatin/NAMPT in RR-MS patients was significantly higher compared those in two other subtypes, whereas, serum leptin levels were significantly decrease in RR-MS patients as compared to SP-MS and PP-MS patients. Also, Foxp3 expression and resistin levels in RR-MS patients were higher compared to other subtypes, although not significantly.

Table 4 shows correlations of studied adipocytokines with levels of TNF-α, IL-1β, hs-CRP, and Foxp3 expression in participants. 

**Table 4:** Correlations of adipocytokines with inflammatory mediators and FoxP3 expression in MS patients and controls.

Variables		Patients			Controls	
		r	p		r	p
Resistin(ng/ml)						
	TNF-α (ng/ml)	0.33	0.02		0.3	0.04
	IL-1β (ng/ml)	0.3	0.02		0.2	0.03
	hs-CRP (mg/l)	0.12	0.04		0.1	0.09
	Foxp3 relative expression	-0.32	0.04		-0.03	0.1
Visfatin(ng/ml)						
	TNF-α (ng/ml)	0.22	0.03		0.21	0.04
	IL-1β (ng/ml)	0.28	0.02		0.04	0.2
	hs-CRP (mg/l)	0.12	0.04		0.1	0.1
	Foxp3 relative expression	-0.34	0.04		0.05	0.2
Leptin(ng/ml)						
	TNF-α (ng/ml)	0.31	0.02		0.3	0.04
	IL-1β (ng/ml)	0.2	0.03		0.2	0.03
	hs-CRP (mg/l)	0.12	0.04		0.1	0.08
	Foxp3 relative expression	-0.32	0.04		-0.02	0.3

Coefficients (r) and p values are calculated by the Pearson correlation model.

TNF-α: tumor necrosis factor-alpha; IL-1β: interleukin-1 beta; hs-CRP: high sensitivity C-reactive protein; Foxp3: forkhead box P3.

All adipocytokines were positively correlated with levels of TNF-α, IL-1β and hs-CRP and negatively correlated with Foxp3 expression in MS patients. In control group, there were positive correlations between circulating leptin and resistin levels with TNF-α and IL-1β, whereas, the serum levels of visfatin were only correlated with levels of TNF-α.

## Discussion

The first observation of this study was a significant elevation in circulating resistin,leptin and visfatin as well as pro-inflammatory mediators in MS patients that was accompanied by markedly decrease in mRNA Foxp3 expression of T cells.

These findings are in accordance with our previous investigations and several other studies indicating an elevation in circulating resistin, leptin, visfatin, TNF-α,IL-1β and hs-CRP as well as decrease in mRNA Foxp3 expression of T cells in MS patients [6,12,17,18]. Also, it has been shown that serum and CSF of twins with MS have higher adiponectin and adipsin concentrations compared with their asymptomatic co-twins [34]. However, some data found no significant difference regarding adipocytokines and pro inflammatory cytokines 

Although, the exact mechanism mediating elevation of these adipokines as well as reduction of mRNA Foxp3 expression in MS remains obscure, one possible explanation for these findings is that increased pro-inflammatory cytokines levels in multiple sclerosis, and the consequent inflammatory milieu could affect adipocytokine levels as well as Treg functions. In this regard, it has been demonstrated that IL-6 had a negative effect on Treg function and also its function abolished at the site of inflammation likely due to decreased levels of IL-2 [35,36].

Thus, in view of these findings, an *in vitro* study has been designed and is currently under investigation in our laboratory, using treatments of PBMC from MS patients with several anti inflammatory agents.

In keeping with previous studies regarding the induction of resistin, visfatin and leptin expression in inflammation-dependent mechanism and also considering these adipocytokines as the putative inflammatory mediators (reviewed in [25,26,31,37]) we found positive correlations between resistin, visfatin and leptin with circulating TNF-α, IL-1β and hs-CRP in MS patients. Hence, it is plausible that high level of these adipocytokines could enhance pro-inflammatory cytokine secretion in a positive feedback loop, which in turn lead to MS.

As regards leptin, our observation contrasts with other studies demonstrating that treatment of PBMC from MS patients with recombinant leptin can induce the secretion of the anti-inflammatory cytokine IL-10 by monocytes and reduce production of pro-inflammatory cytokines such as IFN-γ by T-cells[38].

One explanation of this discrepancy might be due to our *in vivo* study that allows investigating the role of leptin together with other adipocytokines in a complicated signaling network of metabolic and immune systems and provides further insights into the mechanisms by which these mediators contribute to MS.

 Our results also demonstrated positive correlations between circulating visfatin, leptin and resistin levels with some inflammatory mediators in healthy subjects. Therefore, it can be deduced that these adipocytokines function as the possible inducers of pro-inflammatory cytokines even when inflammation is absent. In contrast, Frisullo et al. found that leptin enhances TNF-α IL-6, and IL-10 production from PBMCs of relapsing multiple sclerosis patients, but not in patients in a stable phase or in healthy controls[39]. 

The high levels of circulating adipocytokines in MS patients, on the one hand, and the close interaction between the regulation of body fat and the immune system on the other led us to assess fat distribution by DEXA, in order to identify the possible source of adipocytokines and their functions in inflammation. Although several studies found no significant difference in term of percentage of body fat (%BF) between MS patients and the control subjects [40,41], the present study demonstrated a decreased adipose tissue mass in patients with MS compared with healthy individuals. This observation indicates that complicated pathways might regulate the adipocytokine expression or there are other sources for expression of adipocytokines.

The latter explanation is supported by studies indicating that immune cells such as PBMC and macrophages are now considered as additional sources of adipocytokines [25,31,42], moreover, the participation of T-cells and macrophages resident in adipose tissue [43,44] has not been ignored.

Additionally, the balance between fat deposition and lipolysis has been suggested as a major determinant of fat tissue mass, hence, our finding may partly be explained by a recent study by Mähler et al. [45] in which observed that MS patients had higher lipolytic activity in adipose tissue compared to healthy control subjects.

Furthermore, another possible mechanism for reduced fat tissue mass in MS could be increased circulating pro-inflammatory cytokines including IL-1 β and IL-6 in MS patients. IL-1 β has been shown to inhibit differentiation of preadipocytes into adipocytes [46] ,and to decrease the lipid content in mature adipocytes[47]. Based on several studies in animals and human subjects IL-6 can suppress body fat mass and increase energy expenditure (reviewed in 48).

It also seems likely that cross-talk between inflammation and Treg functions plays an important role in the pathogenesis of MS. In this regard, our study has demonstrated significant and inverse correlations between circulating concentration of leptin, visfatin and resistin with Foxp3 ( the master gene for Treg cell development and function) in MS patients. This observation concerning leptin was in line with previous reports demonstrating that there is an inverse correlation of this adipocytokine with frequency of Tregs and also the mean fluorescence channel (MFC) of Foxp3 in RR-MS patients[16,18].

More importantly, to date, no evidence exists so far with regards to the correlation between resistin and visfatin with regulatory T cell homeostasis (manifested by alteration in Foxp3 expression) in patients suffering from inflammatory and autoimmune disease. For this reason, our findings strengthen the concept that other adipocytokines, in addition to leptin function as negative signals for the expansion of human naturally occurring Foxp3^+^ CD4^+^CD25^high^ regulatory T cells [49]. As reviewed in detail elsewhere [9], the inflammatory environments could lead to the loss of Foxp3 expression in the Treg cells, thereby contributing to dysregulation of these cells. 

More recently it has been shown that the mice deficient for the NAD-hydrolase CD38, had lower Treg cell numbers in comparison with wild type [50]. Thus, the possible mechanism underlying the negative correlation of visfatin with Foxp3 might be due to the function of visfatin in the initiation of *de novo* NAD biosynthesis and influence of NAD on Treg function and cell viability.

Considering that MS is a highly heterogeneous disease in term of clinical course and immunopathogenesis, it seems necessary to divide the MS patients into their different clinical courses. This study showed markedly higher level of inflammatory markers in PP-MS patient compared to RR-MS and SP-MS patients, which is in agreement with some data showed dysregulated inflammatory and immune responses could contribute to the observed clinical course of MS [6,51,52]. However, some studies provided evidence to indicate that there is no difference in this regard [53]. In addition, the current study demonstrated increased, but not significantly expression of Foxp3 in patients with RR-MS in comparison with patients with SP-MS and PP-MS. Recently, it has been demonstrated that the Foxp3 expression from CD4^+^CD25^-^ T-cell pool of MS patients was inversely correlated with the production of IFN-γ, IL-6, IL-21 and IL-17 and also IL-6 neutralization of the culture of PBMC from MS patients markedly enhanced the expression of FoxP3 in FoxP3^low^ T-cell clones[54] ,therefore, our observation can be interpreted in light of the inflammatory nature of MS.

Confirming evidence mentioned, the highest levels of TNF-α, IL-1β, hs-CRP, resistin and leptin as putative inflammatory mediators , were observed in PP-MS patients presenting with lowest Foxp3 expression. However, in contrast to our findings, Venken K *et al.* [55] showed that patients with RR-MS had reduced Foxp3 expression level and lower numbers of circulating CD4_+_ CD25^high^ Foxp3^+^ T cells compared with SP-MS patients. More importantly, our study shows that patients with PP-MS had the lowest concentration of visfatin compared to other MS subtypes as well as the highest level of resistin and leptin, although, this difference did not reach statistical significance for resistin.

As regards visfatin, this is partly in line with a recent study by Mirzaei K et al [17] in which observed that its level tended to be higher, albeit not significantly in patients with RR-MS as compared with those in SP-MS and PP-MS as a single group. Although the exact mechanism underlying the transition from the relapsing remitting to the progressive phase of MS is unclear, epidemiological and MRI studies indicate that the neurodegenerative pathological changes seems to underlie this evolution[56]. In view of increase in vulnerability of neurons to degeneration upon NAD deficiency [57] and prevention of degeneration of transected axons following exogenous application of NAD or nicotinamide [58], the neuroaxonal degeneration observed in multiple sclerosis with PP-MS and SP-MS may be partly related to reduction of visfatin/NAMPT as a rate-limiting enzyme generating NAD.

While numerous other candidate biomarkers of disease activity in serum and cerebrospinal fluid of MS patients have been described, none so far have demonstrated sufficient clinical utility.

Accordingly, our study provides novel insights into adipocytokines as the relative biomarkers in different clinical subtypes of MS, which in absence of the unique, definitive and specific biomarkers might be helpful. In addition, another advantage of the current study is simultaneous measurements of several adipocytokines for the first time and the evaluation their cross-talk with key inflammatory mediators and Foxp3 expression in different MS subtypes.

However, more investigation and large-scale monitoring is required to clarify whether the changes in circulating adipocytokines precedes transitions from a purely relapsing into a progressive phase of disease or not. Another limitation of this study is the lack of specificity of adipocytokines that may undermine their utility as definitive biomarkers in MS. Accordingly, the measurement of additional adipocytokines and immune-related markers warrant further consideration for multifaceted and comprehensive assessment of MS.

 In addition, this study was limited by the analysis of Foxp3 mRNA expression by quantitative real-time PCR, thus, a more quantitative and sensitive analysis of the phenotypic and functional characteristics of CD4^+^CD25^+^ regulatory T cells by flow cytometry would be useful.

Collectively, our data show that the assessment of adipocytokines together with other inflammatory cytokines pattern could be partially useful to elucidate complex and heterogenous pathophysiology of MS. Furthermore, our observations suggest that not only a dysregulated and complex network of mediators encompassing cytokines, immune cell subsets and adipocytokines, but also their cross-talk are involved in the pathogenesis of MS. However, further studies are needed to clarify whether the assessment of alteration of the adipocytokine pattern together with other immunological and inflammatory factors can be considered as reliable biomarkers helping to elucidate different clinical features in MS or not.

## Conclusion

**Figure 1:**
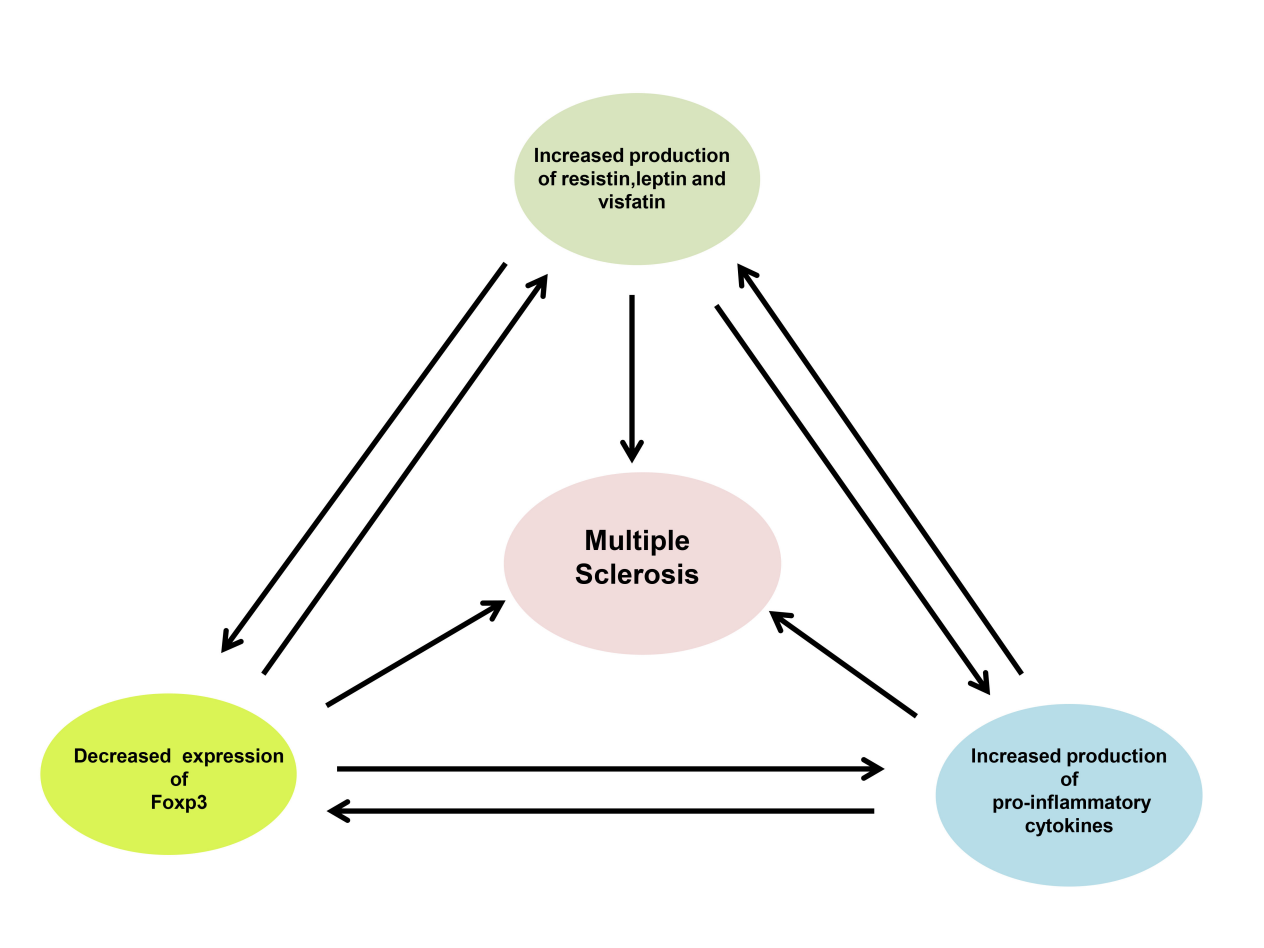
Schematic diagram of the proposed mechanism for the pathogenesis of MS. The elevation of circulating adipocytokines and pro-inflammatory mediators on one hand, and the reduction of FoxP3 expression on the other hand have provided strong evidences into understanding the pathogenesis of MS. Additionally, it is plausible that high level of resistin, visfatin and leptin could enhance pro-inflammatory cytokine secretion in a positive feedback loop, which in turn lead to the loss of Foxp3 expression.

**Figure 2:**
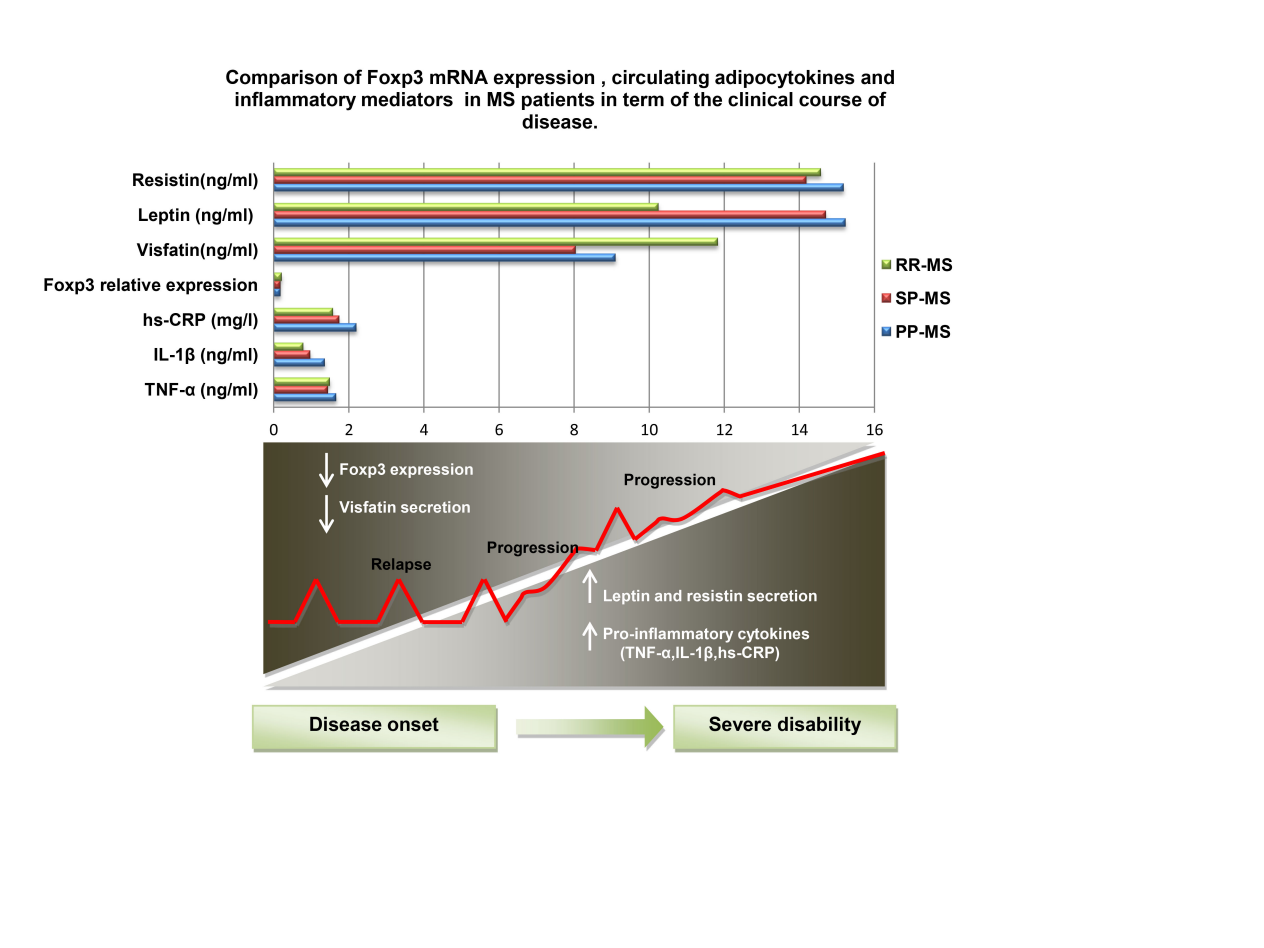
Comparison of Foxp3 mRNA expression and circulating adipocytokines and inflammatory mediators in MS patients in term of the clinical course of disease. In parallel with disease progression in patients with MS, the level of inflammatory markers as well as adipocytokines including resistin and leptin decreased and the expression of FoxP3 and circulating level of visfatin increased. TNF-α: tumor necrosis factor-alpha; IL-1β: interleukin-1 beta; hs-CRP: high sensitivity C-reactive protein; Foxp3: forkhead box P3.

In conclusion, we present novel data of increased serum adipocytokines and pro-inflammatory mediators, despite decreased adipose tissue mass as well as decreased FoxP3 expression in MS patients compared to healthy individuals. More importantly, we demonstrated that all studied adipocytokines negatively associated with Foxp3 expression and positively correlated with levels of inflammatory mediators (Figure 1). In addition, the major new finding of this study is that in parallel with disease progression in patients with MS, the level of inflammatory markers as well as adipocytokines including resistin and leptin decreased and the expression of FoxP3 and circulating level of visfatin increased. (Figure 2)
