# Correction: Adipocytokine Profile, Cytokine Levels and Foxp3 Expression in Multiple Sclerosis: a Possible Link to Susceptibility and Clinical Course of Disease

**DOI:** 10.1371/annotation/6de84c11-73f5-4ee4-aec7-ca83072401a4

**Published:** 2013-11-07

**Authors:** Solaleh Emamgholipour, Seyede Mahdieh Eshaghi, Arash Hossein-nezhad, Khadijeh Mirzaei, Zhila Maghbooli, Mohammad Ali Sahraian

Affiliation number 4 for the fourth author, Khadijeh Mirzaei, is incorrect. The correct affiliation is: Department of Nutrition and Biochemistry, School of Public Health and Institute of Public Health Research, Tehran University of Medical Sciences, Tehran,Iran. 

