# Correction: Adipocytokine Profile, Cytokine Levels and Foxp3 Expression in Multiple Sclerosis: a Possible Link to Susceptibility and Clinical Course of Disease

**DOI:** 10.1371/annotation/8fc7e49e-2790-4cfa-900c-2a1e319d61b8

**Published:** 2014-01-06

**Authors:** Solaleh Emamgholipour, Seyede Mahdieh Eshaghi, Arash Hossein-nezhad, Khadijeh Mirzaei, Zhila Maghbooli, Mohammad Ali Sahraian

The second sentence of the Conclusion is incorrect. The second sentence should read: "In addition, the major new finding of this study is that in parallel with disease progression in patients with MS, the level of inflammatory markers as well as adipocytokines including resistin and leptin increased and the expression of FoxP3 and circulating level of visfatin decreased."

In addition, the title and legend for Figure 2 are incorrect. The correct title is: "**Comparison of Foxp3 mRNA expression and circulating adipocytokines and inflammatory mediators in MS patients in term of the clinical course of disease.**" The correct legend is: "In parallel with disease progression in patients with MS, the level of inflammatory markers as well as adipocytokines including resistin and leptin increased and the expression of FoxP3 and circulating level of visfatin decreased. TNF-α: tumor necrosis factor-alpha; IL-1β: interleukin-1 beta; hs-CRP: high sensitivity C-reactive protein; Foxp3: forkhead box P3. doi:10.1371/journal.pone.0076555.g002"

